# Fuzzy nanostructure growth on Ta/Fe by He plasma irradiation

**DOI:** 10.1038/srep30380

**Published:** 2016-07-25

**Authors:** Shin Kajita, Tomoya Ishida, Noriyasu Ohno, Dogyun Hwangbo, Tomoko Yoshida

**Affiliations:** 1 Institute of Materials and Systems for Sustainability, Nagoya University, Nagoya 464-8603, Japan; 2 Graduate School of Engineering, Nagoya University, Nagoya 464-8603, Japan; 3 The Osaka City University Advanced Research Institute for Natural Science and Technology, Osaka 558-8585, Japan

## Abstract

In this study, we show from helium (He) plasma irradiation to tantalum and iron surfaces that morphology changes in nanoscale occur on the both metals. In particular, from systematic irradiation experiments, it is identified that fuzzy nanostructures are grown on the both metals. The necessary conditions for the morphology changes are discussed based on the experimental results in terms of the helium migration, the physical sputtering, and the shear modulus of materials. Because oxides or oxinitrides of iron and tantalum are thought of as visible light responsive photocatalytic materials, the present work shows wide potential of usage of plasmas as a tool to tailor photocatalytic materials.

One of efficient usages of the solar light is the production of hydrogen by photocatalytic reactions. Although titanium oxide (TiO_2_) is a well-known photocatalytic material for water splitting^1^, it can only utilize ultraviolet (UV) wavelength ranges for the reaction. Since the fraction of the UV range in the solar light is 3–5%, various studies have been conducted to use longer wavelength ranges for water splitting^2^. Oxides or (oxy)nitrides of various metals such as tungsten (W)^3^, iron (Fe)^4^, niobium (Nb)^5^, and tantalum (Ta)^6^,^7^ are candidate materials for visible light responsive photocatalysts. However, photocatalyst with enough efficiency for practical application has yet to be achieved.

To tailor highly reactive photocatalytic materials, a large effective surface area with a high porosity is one of the most important factors. Three dimensionally porous structures including voids are of importance, while periodicity is not required^8^. It has been found that a meso-porous Nb_2_O_5_ had 20 times higher photocatalytic activity than a bulk one without any porosity^5^. Recently, helium (He) plasma irradiation has been used to fabricate meso-porous fiberform nanostructure, so-called fuzz, on various metals including W^9^,^10^,^11^, molybdenum (Mo)^11^,^12^, Ti^13^, Fe^14^,^15^, nickel (Ni)^14^,^16^, rhenium (Re)^16^, and platinum (Pt)^17^. Also, it was revealed that He plasma irradiation to Ta^16^,^18^ and Nb^19^ changes the surface morphology in nanometer scale, though no fiberform structure has been developed. On aluminum and copper, formations of voids and nano pillars have been observed by He plasma irradiation^20^. In addition to the fact that the nanostructured metals become almost totally solar light absorber^21^ with a high effective surface area^22^, they have inner voids and porous structure in addition to outer fiberform structures. Thus, there is a potential that the He irradiated metals can be highly active photocatalytic material.

Photocatalytic activities of oxidized He irradiated W have been investigated. It was revealed that oxidized He irradiated W increased the efficiency of water splitting^23^. From methylene blue de-colorization experiments, it was shown that partially oxidized He irradiated W has high visible light reactivity^14^,^24^. It is of interest to further explore possibilities of the usage of plasmas for tailoring highly reactive photocatalytic materials.

In this study, we will explore the possibility of plasma irradiation on the surface morphology changes of Ta and Fe, which can be utilized for photocatalytic materials. Different from previous studies^15^,^16^,^18^,^19^, fiberform nanostructures were formed on Ta and Fe; differences in the experimental conditions will be discussed. Based on the experimental results, a comprehensive discussion about the fuzzy nanostructure growth on metals is given in terms of the effects of physical sputtering, He migration, and shear modulus of materials.

## Methods

Experiments were conducted in the linear plasma device NAGDIS-II (Nagoya Divertor Simulator). High density plasmas, typically 10^18^–10^19^ m^−3^, can be produced in steady state. Samples (Ta and Fe) were mounted on a water cooling stage and exposed to the plasma in the downstream region of the device. The size of the sample was 10 × 10 mm and thickness was typically 0.2 mm. The incident ion energy was controlled by changing the voltage of the sample. The temperature of the sample was measured with a radiation pyrometer.

The formation of fiberform nanostructures by He irradiation on metals is an common phenomenon observed in various devices worldwide for W and Mo. It is noted that same morphology changes were identified on low-flux devices^25^,^26^ including magnetron sputtering devices in addition to linear devices that can produce rather high density plasmas. It was found from experiments for W that the important parameters for the nanostructure growth are the incident ion energy (>20–30 eV), the surface temperature (1000–2000 K), and the He fluence (>10^25^ m^−2^)^11^. In this study, we conducted He plasma irradiation experiments with changing those parameters.

After the He irradiation, the surface was observed by scanning electron microscopy (SEM), and the optical reflectance (total) was measured by a spectrophotometer (UV-2600, Shimadzu, Co.). The specular and diffusive reflections were collected with an equipped small integrating sphere. The incident angle of light beam to the sample was 8 degree from the normal angle.

## Results

### Tantalum

**Figure 1:**
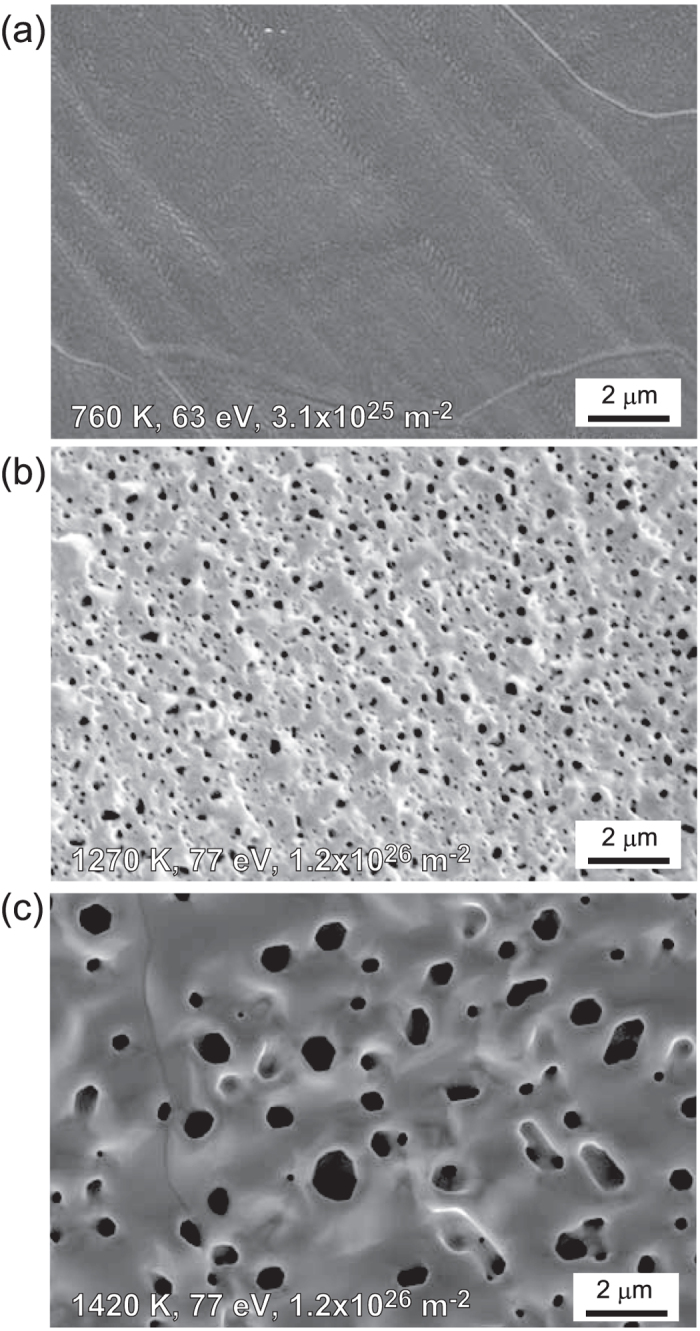
SEM micrographs of Ta samples exposed to the He plasmas at the surface temperatures of (**a**) 760, (**b**) 1270, and (**c**) 1420 K. The incident ion energy and the He fluence were (**a**) 63 eV and 3.1 × 10^25^ m^−2^, respectively, and (**b**,**c**) 77 eV and 1.2 × 10^26^ m^−2^, respectively.

Figure 1(a–c) show SEM micrographs of Ta samples exposed to the He plasmas at different surface temperatures of 760, 1270, and 1420 K, respectively. The incident ion energy was (a) 63 eV and (b,c) 77 eV, and the He fluence was (a) 3.1 × 10^25^ and (b,c) 1.2 × 10^26^ m^−2^. When the surface temperature was 760 K, no significant change was observed on the surface, while pinholes cover the surface when the temperature is higher (1270 and 1420 K). The size of the pinholes increased with the surface temperature.

**Figure 2:**
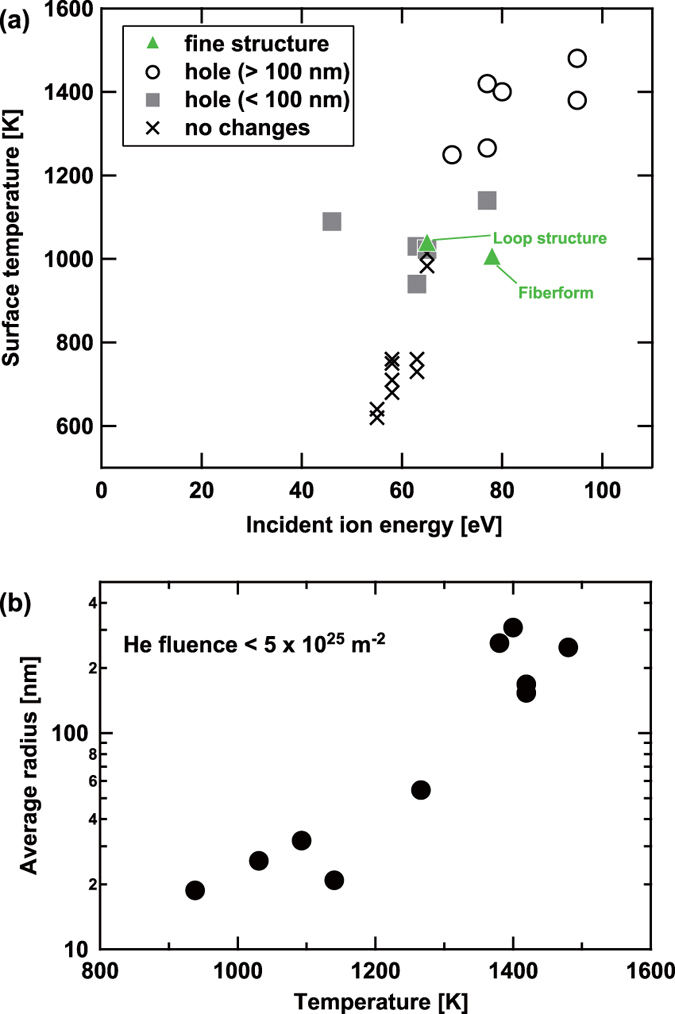
(**a**) A summary of the surface structural changes and the irradiation conditions, i.e. the surface temperature and the incident ion energy, and (**b**) the averaged radius of the pinholes as a function of the surface temperature.

Figure 2(a) summarizes the surface structural changes and the irradiation conditions, i.e. the surface temperature and the incident ion energy. In the energy range used in this study (45–95 eV), no clear incident ion energy dependence was identified, though a clear dependence in the surface temperature was seen. No pinholes were formed on the surface when the surface temperature was less than 800 K. On the other hand, when the surface temperature was higher than 900 K, many pinholes were found on the surface. Moreover, with increasing the surface temperature, the size of the pinholes increased. In Fig. 2(b), the averaged radius of the pinholes is shown as a function of the surface temperature. The averaged radius became greater than 100 nm when the surface temperature was higher than 1200 K.

Previously, it was observed that the size of the pore formed on the surface increased with the surface temperature^18^ even when the fluence was on the order of 10^24 ^m^−2^. Basically, the tendency observed in the present study was the same as the one done previously. However, difference was identified in the size of pinholes in higher temperatures; the size becomes significantly greater than the typical size in the low fluence case (~70 nm^2^) when the temperature is higher than 1200 K.

**Figure 3:**
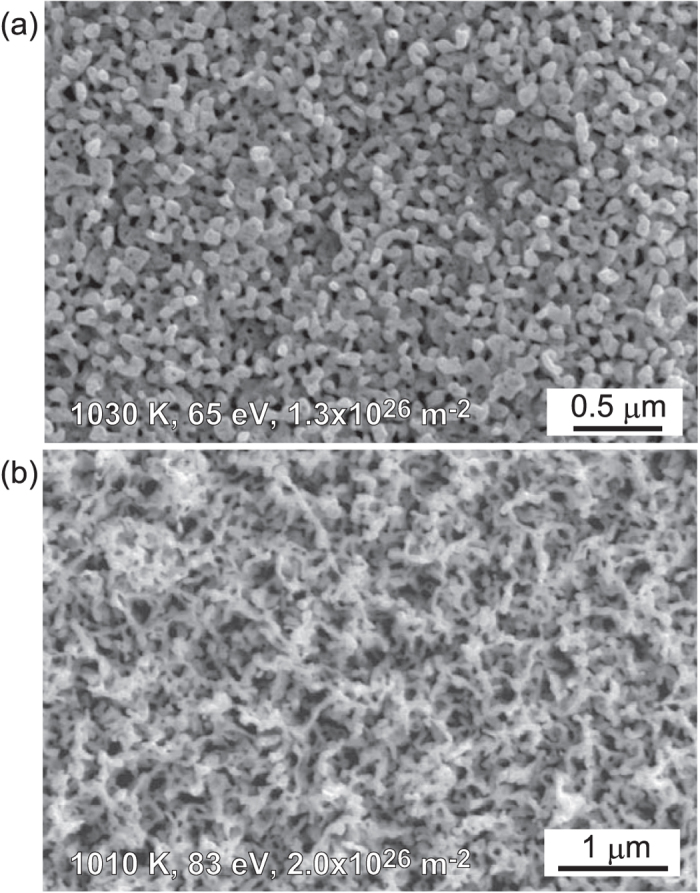
SEM micrographs of the He irradiated Ta surfaces. The irradiation conditions (the incident ion energy, surface temperature and He fluence) are (**a**) 65 eV, 1030 K, and 1.3 × 10^26^ m^−2^ and (**b**) 83 eV, 1010 K, and 2.0 × 10^26^ m^−2^.

We explored higher fluence irradiation with the fluence of the order of 10^26^ m^−2^ and investigated the surface structural changes by the irradiation. Figure 3(a) shows an SEM micrograph of the He irradiated surface at the incident ion energy and irradiated surface temperature of 65 eV and 1030 K, respectively, with the He fluence of 1.3×10^26^ m^−2^. It is seen that a rough surface with finer loop-like structures was formed on the surface. Figure 3(b) shows an SEM micrograph of the sample at slightly higher incident energy of 83 eV. The irradiated surface temperature was 1010 K and the fluence was 2.6×10^26^ m^−2^. It was found that fiberform nanostructures were formed on the surface.

### Iron

**Figure 4:**
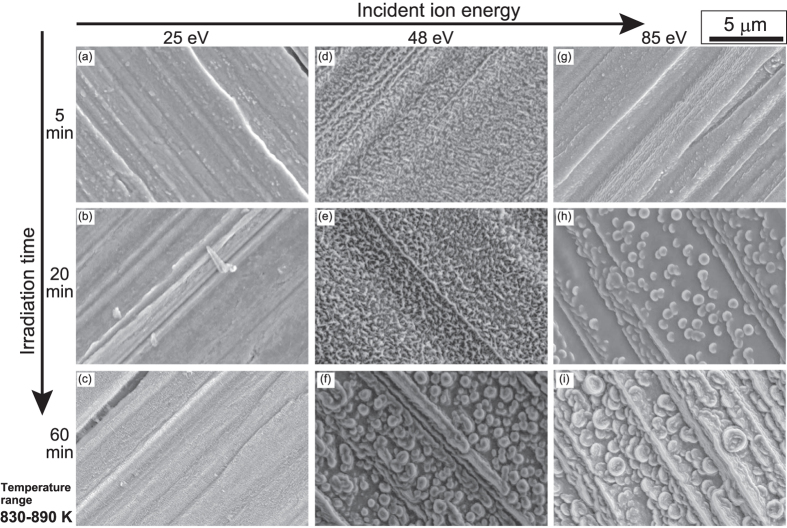
SEM micrographs of Fe surfaces irradiated with He plasmas. The incident ion energies were (**a**–**c**) 25 eV, (**d**–**f**) 48 eV, and (**g**–**i**) 85 eV, and the irradiation times were (**a**,**d**,**g**) 5 min, (**b**,**e**,**h**) 20 min, and (**c**,**f**,**i**) 60 min. The surface temperature during the irradiation was in the range of 830–890 K: (**a**) 880, (**b**) 840, (**c**) 840, (**d**) 850, (**e**) 860, (**f**) 830, (**g**) 880, (**h**) 890 and (**i**) 870.

First, we conducted irradiation experiments in the temperature range of 830–890 K by changing the fluence, i.e., the irradiation time and incident ion energy. Figure 4 shows the SEM micrographs of Fe surfaces irradiated with He plasmas. In Fig. 4, the incident ion energies were (a–c) 25 eV, (d–f) 48 eV, and (g–i) 85 eV, and the irradiation times were (a,d,g) 5 min, (b,e,h) 20 min, and (c,f,i) 60 min. The He flux was in the range of 1.1–4.7×10^22 ^m^−2^s^−1^. When the incident ion energy was 25 eV, no significant morphology changes were observed.

**Figure 5:**
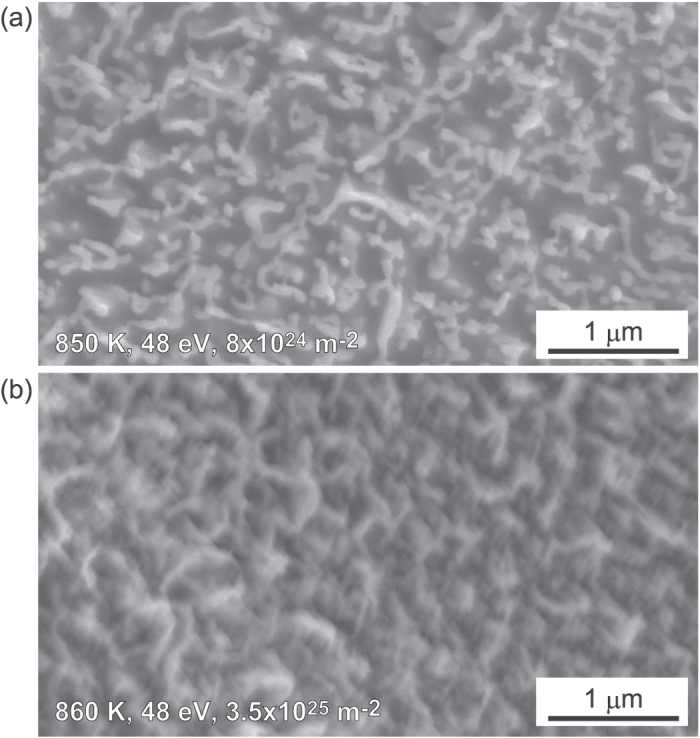
SEM micrographs of the He irradiated Fe surfaces at 48 eV for (**a**) 20 and (**b**) 60 min, shown in Fig. 4(c,d), respectively, with higher resolution.

When the incident ion energy was increased to 48 eV or higher, the surface became rough in micro-scale, and the roughness was enhanced with increasing the irradiation time. Figure 5(a,b) show enlarged SEM micrographs of Fig. 4(d,e), respectively. When the incident ion energy was 48 eV, fiberform structures were started to be grown on the surface. On iron, it is noted that pinholes were not found on the surface. Moreover, the growth process of microstructures found on Fig. 4(f,h,i) is different from the growth of fiberform structures on W and Mo discussed in^11^,^16^,^27^. It seems that surface was swollen similar as blisters formed when irradiating hydrogen plasmas on W^28^. At the moment, it is not obvious why the growth of fiberform nanostructures was not identified at 60 min cases, though some initial growth was observed at the incident ion energy was 48 eV for 5 and 20 min irradiation. It is likely that the growth of fiberform nanostructures is not stable in this temperature range probably by competing with other processes. One possible process is physical sputtering, as discussed later.

**Figure 6:**
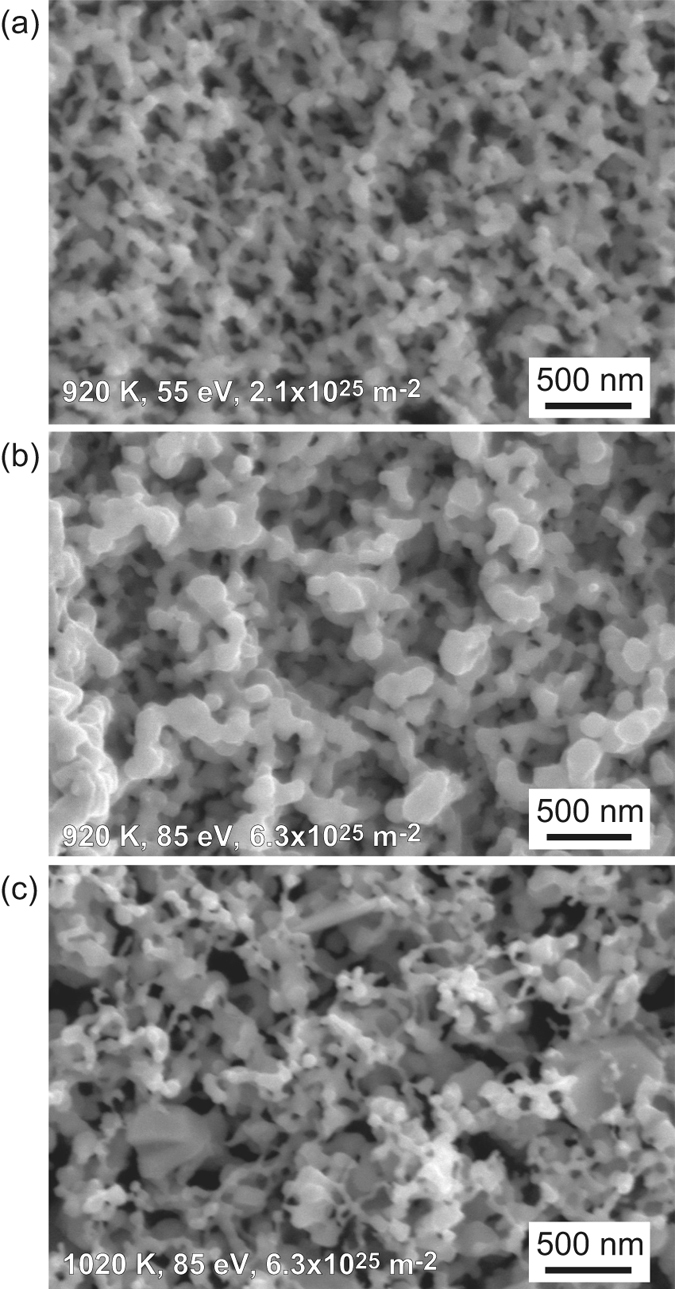
SEM micrographs of Fe surfaces exposed to He plasma irradiation at the surface temperature of (**a**) 920, (**b**) 920, and (**c**) 1020 K. The incident ion energy and He fluence were (**a**) 55 eV and 2.1 × 10^25^ m^−2^, respectively, (**b**) 85 and 6.3 × 10^25^ m^−2^, respectively, and (**c**) 85 eV and 6.3 × 10^25^ m^−2^, respectively.

When the surface temperature was raised slightly to higher than 900 K, further growth of fiberform nanostructures is observed. Figure 6(a–c) show SEM micrographs of Fe surfaces exposed to He plasma at the surface temperature of 920, 920, and 1020 K, respectively, and the incident ion energy of 55, 85, and 85 eV, respectively, up to the He fluence of 2.1, 6.3, and 6.3 × 10^25^ m^−2^, respectively. On the surface in Fig. 6(c), larger blocks were observed beneath the fiberform structures, and diameter becomes larger around the top layer in Fig. 6(b). It should be mentioned that significant morphology changes did not occur when the surface temperature and the incident ion energy were 1020 K and 55 eV, respectively, with the similar He fluence, though we did not show the SEM here. It is likely that there is an upper temperature limit around 1000 K for the fine morphology changes.

## Discussion

Previously, from He plasma irradiation to Ta, it was reported that fuzz was not grown on the surface^16^,^18^. In ref. 18, no further significant morphology changes from pinholes were observed on Ta. Takamura *et al.* reported that loop-like rough surfaces were observed on Ta in addition to pinholes^16^. In those previous studies, the He fluence on Ta was less than ~2 × 10^25^ m^−2^. The He fluence in the present study was one order of magnitude greater when the nanostructures were formed on the surface. In ref. 16, it was discussed from the relation between the temperature dependences of the shear modulus and He mobility that the formation of the fiberform structures does not easily occur on Ta compared with W and Mo. Although the window of the formation condition might be narrower, it was confirmed that the nanostructures could be formed on Ta surface as well by He plasma irradiation. From the present experiments, it can be said that much higher He fluence, typically one order of magnitude higher than that for W, is required for the growth of Ta nanostructures.

Concerning Fe, previously, temperature and fluence dependences of the morphology change of Fe surfaces by He plasma irradiation have been investigated at the incident ion energy of 25 eV in the surface temperature range of 750–970 K in pilot-PSI^15^. It was reported that the surface became porous in nanoscale, though the structure was different from fuzzy nanostructures; in the present study, no significant morphology changes were observed on the surface when the incident ion energy was 25 eV. A clear difference in the experimental condition was in the He flux. In ref. 15, the He flux was 3.5–6.5 × 10^23^ m^−2^s^−1^, which was 8–60 times greater than that in the present experiments. Similar to the He flux threshold observed on the formation of W nanostructures^29^, a He flux threshold may exist on the structural change when the incident ion energy was around 25 eV. Considering the fact that fuzzy fiberform structures were also not grown in previous study when the incident ion energy was 25 eV, it is likely that there is a energy threshold between 25–50 eV for the formation of nanostructures on iron, similar to W, which has an energy threshold around 20–30 eV^11^.

**Figure 7:**
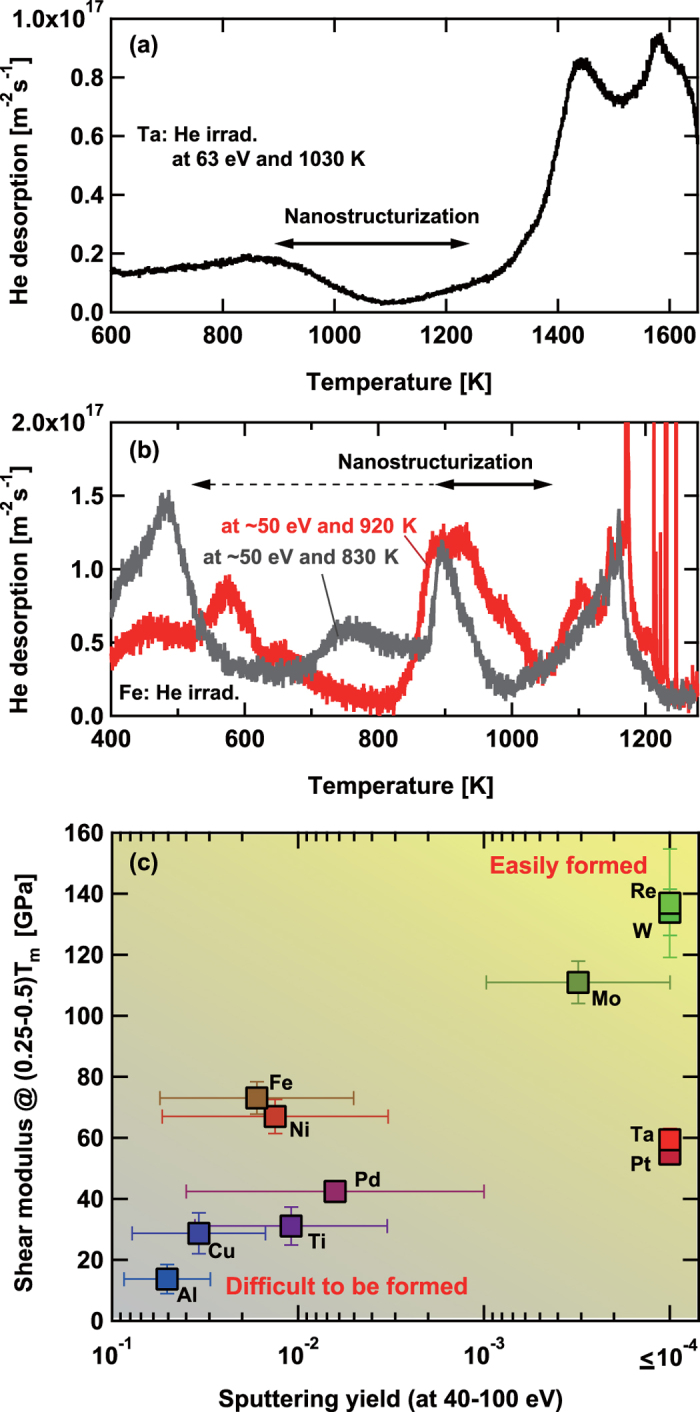
TDS spectra from (**a**) a Ta sample and (**b**) Fe samples exposed to the He plasmas. (**c**) shows the shear modulus (vertical axis) and sputtering yield (horizontal axis) for various metals. The vertical error bar represents the maximum and minimum of the shear modulus in the temperature range from 0.25*T*_m_ to 0.5*T*_m_, and the horizontal error bar represents the maximum and minimum sputtering yield in the range of 40–100 eV.

The surface temperature is an important parameters for the nanostructure growth. It significantly changes the migrations of He atoms/clusters/bubbles inside the metal and the growth rate of the He bubbles. To check the behavior of He inside the metal, thermal desorption spectroscopy (TDS) analysis was performed on He irradiated Ta and Fe samples. In general, samples exposed to He ions have broader desorption spectra compared with samples exposed to hydrogen isotopes. This is because various processes can occur with increasing the temperature such as growth of loop punching and inter-bubble fracture, coalescence of He bubbles, formation of blisters, and migration of bubbles to the surface^30^. Although the confirmation of processes corresponding to the desorption spectra may be difficult, the He desorption spectra can be helpful to understand the mobility of the He in the sample^13^. Figure 7(a) shows the TDS spectra from Ta sample exposed to the He plasma at the incident energy of 63 eV and the surface temperature of 1030 K. A small peak appeared at 850 K, and a significant He desorption was identified after a dip around 1100 K when the surface temperature was higher than 1300 K. The TDS spectrum is consistent with the morphology changes: no pinhole formation below the lower peak of ~850 K and significant increase of pinhole size higher than 1200–1300 K. Figure 7(b) shows the TDS spectra from two Fe samples exposed to the He plasma at the incident ion energy of 50 eV and different surface temperatures (830 and 920 K). We can identify the minimum desorption peak at ~500–600 K and the maximum desorption peak at ~1150 K. This indicates that the surface morphology changes can occur when the surface temperature is higher than 500–600 K, as shown previously in ref. 15, but the He migration rate becomes too much if the temperature is close to the maximum peak at 1150 K. Different from Ta, other desorption peaks were also identified between the minimum and maximum peaks. This suggested that the morphology changes will be enhanced when the temperature is higher than 900 K, where we can identify a desorption peak. This is consistent with the experimental results shown in Figs 4, 5, 6. Short whiskers have been observed when the surface temperature was <900 K, but significant nanostructure growth was only identified at higher temperatures of 920 and 1020 K.

Different from W and Mo, sputtering can play significant role for the morphology changes on other metals. Concerning Ta, similar to W and Mo, the sputtering yield is considerably low, say <10^−4^, when the energy is lower than 100 eV^31^. However, for Fe, the influence of sputtering cannot be neglected. The sputtering yield is ~10^−2^ at the incident ion energy of 48 eV and couple of times greater at 85 eV. If the growth rate of the nanostructure of Fe is same as that on W, thin nanostructure layer less than 1 *μ*m can be formed when the sputtering yield is on the order of 10^−2 ^32. However, if the growth rate is smaller and the sputtering yield is greater, there would be no chance for the nanostructures to be grown on the surface, though it may be possible to see short whiskers on the surface. In Fig. 4, nanostructures were not identified when larger blister like structures were seen on the surface. From a previous study on Ti, of which the sputtering yield is similar to that of Fe (10^−2^ at 50 eV)^31^, fiberform nanostructures were rarely identified, though pinholes, cone structure, and larger bubbles were formed^13^. One of the possible reasons to explain such unstable fuzz growth on both Fe and Ti is an increase of sputtering due to the angular dependence^31^. When larger structural changes occur, the particles bombard the surface in shallower angles, and the sputtering yield may increase, because it increases in general with decreasing the incident angle. The angular dependence may change the balance between the nanostructure growth and the sputtering especially when the sputtering is originally significant (typically when the sputtering yield is >10^−2^).

One of the other important parameters is likely the shear modulus^16^. It was discussed that there may be a low limit in the shear modulus for the nanostructure growth. In Fig. 7, the shear modulus and the sputtering yield for various metals are summarized. Because the fuzzy nanostructures were identified in the temperature range of 0.25–0.5*T*_m_^16^, where *T*_m_ is the melting point, the shear modulus in the temperature range was plotted using error bars by considering the temperature dependence^33^,^34^,^35^,^36^,^37^. Although the shear modulus is also influenced by the He concentration^38^, the values without He can be an index to see the tendency of the nanostructure growth. Concerning the sputtering yield, the maximum and minimum sputtering yields in the range of 40–100 eV^31^,^37^ are shown using error bars. It is seen that W and Mo are plotted in the upper right of the figure, where the nanostructures are likely to be formed easily. On the other hand, the conditional window for the fuzzy nanostructures would be narrow on the materials plotted in the other part of the figures, especially, in the lower left corner.

**Figure 8:**
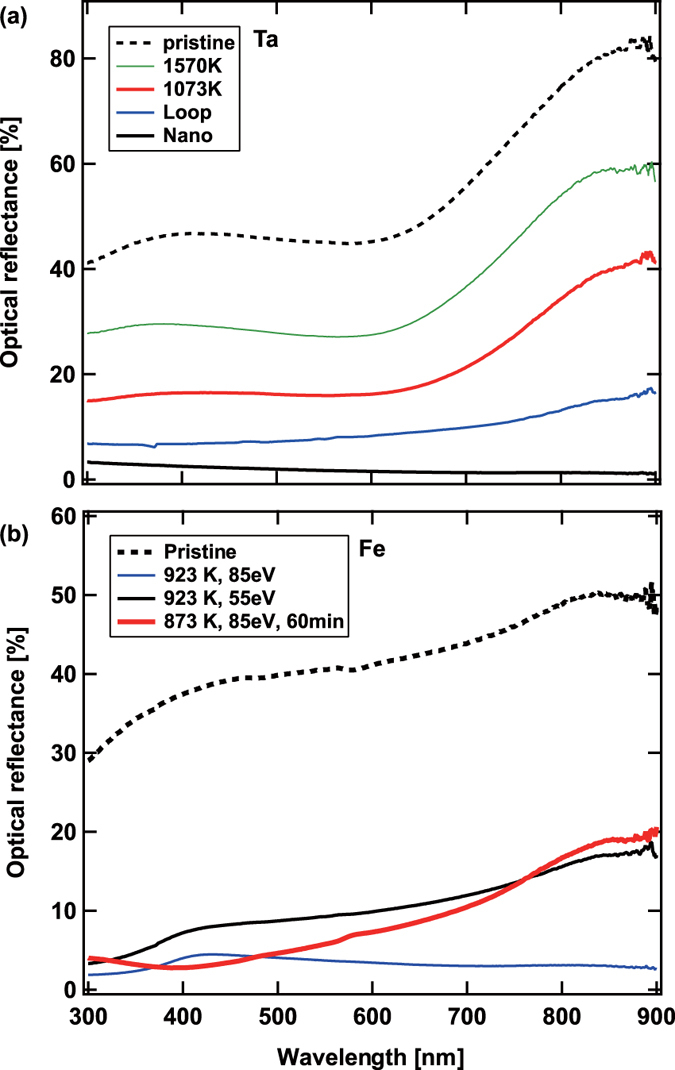
The wavelength dependences of the optical reflectance of (**a**) Ta and (**b**) Fe samples exposed to the He plasmas.

Figure 8(a) shows the wavelength dependences of the optical reflectance of a pristine Ta sample and Ta samples exposed to the He plasmas. The optical reflectance decreased by the He plasma irradiation. Concerning the samples with pinholes, when the surface temperature was rather high (1570 K), the optical reflectance was decreased by typically 25–30%, while when the irradiation temperature was lower (1073 K) and the pinhole size was smaller, the optical reflectance decreased to roughly half before the irradiation. On the sample with loop-like fine structures, the reflectance decreased to ~20% of that before the irradiation. When the nanostructures were formed, the optical reflectance was significantly low, typically ~1%, though it slightly increased to several % with decreasing the wavelength. Figure 8(b) shows the wavelength dependences of the optical reflectance of iron samples. The reflection properties of three samples exposed to the He plasmas shown in Figs 4(i) and 6(a,b) and a pristine sample are shown. The reflectance decreased by the He plasma irradiation on all the three samples as well. Especially, the sample shown in Fig. 6(b) had low reflectance less than 5%, which is approximately one order of magnitude less than that of pristine, in the wavelength of 300–900 nm. Since the surface was not oxided, nitrided, or oxynitrided, the absorptance does not necessarily reflect the photocatalytic activity; it is necessary to conduct experiments to measure the photocatalytic behavior for future work. Here, the increase in the optical absorptivity indicates a possibility that the efficiency of photocatalytic activity can be increased by an increase in the effective surface area. For the case of W samples, when the optical reflectance was ~1% at 633 nm, the layer was ~500 nm^21^, where the effective surface area from Brunauer-Emmett-Teller (BET) analysis method using gas (krypton) absorption on the surface was measured to be one order of magnitude greater than that of a pristine sample^22^. Similarly, it is expected that the nanostructured Ta surface has approximately one order of magnitude greater effective surface area and has higher photocatalytic activity when the surface is nitrided or oxynitrided. Also, the effective surface area of the He irradiated Fe should be increased more than severalfold.

## Additional Information

**How to cite this article**: Kajita, S. *et al.* Fuzzy nanostructure growth on Ta/Fe by He plasma irradiation. *Sci. Rep.* **6**, 30380; doi: 10.1038/srep30380 (2016).

## References

[b1] Fujishima A. & Honda K.. Electrochemical photolysis of water at a semiconductor electrode. Nature 238, 37–38 (1972). 12635268 10.1038/238037a0

[b2] Chen X., Shen S., Guo L. & Mao S. S.. Semiconductor-based photocatalytic hydrogen generation. Chemical Reviews 110, 6503–6570, pMID: 21062099 (2010). 21062099 10.1021/cr1001645

[b3] Zhao Z.-G. & Miyauchi M.. Nanoporous-walled tungsten oxide nanotubes as highly active visible-light-driven photocatalysts. Angewandte Chemie International Edition 47, 7051–7055 (2008). 10.1002/anie.200802207 18663704

[b4] Seki M., Yamahara H. & Tabata H.. Enhanced photocurrent in rh-substituted –Fe_2_O_3_ thin films grown by pulsed laser deposition. Applied Physics Express 5, 115801 (2012).

[b5] Chen X. *et al.* Enhanced activity of mesoporous Nb_2_O_5_ for photocatalytic hydrogen production. Applied Surface Science 253, 8500–8506 (2007).

[b6] Hitoki G. *et al.* An oxynitride, taon, as an efficient water oxidation photocatalyst under visible light irradiation. Chem. Commun. 16, 1698–1699 (2002). 10.1039/b202393h 12196955

[b7] Takata T. *et al.* Visible-light-driven photocatalytic behavior of tantalum-oxynitride and nitride. Research on Chemical Intermediates 33, 13–25 (2007).

[b8] Rolison D. R.. Catalytic nanoarchitectures-the importance of nothing and the unimportance of periodicity. Science 299, 1698–1701 (2003). 12637736 10.1126/science.1082332

[b9] Takamura S., Ohno N., Nishijima D. & Kajita S.. Formation of nanostructured tungsten with arborescent shape due to helium plasma irradiation. Plasma Fusion Res. 1, 051 (2006).

[b10] Baldwin M. & Doerner R.. Helium induced nanoscopic morphology on tungsten under fusion relevant plasma conditions. Nucl. Fusion 48, 035001 (5pp) (2008).

[b11] Kajita S., Sakaguchi W., Ohno N., Yoshida N. & Saeki T.. Formation process of tungsten nanostructure by the exposure to helium plasma under fusion relevant plasma conditions. Nucl. Fusion 49, 095005 (2009).

[b12] Temmerman G. De *et al.* Nanostructuring of molybdenum and tungsten surfaces by low-energy helium ions. Journal of Vacuum Science & Technology A 30, 041306 (2012).

[b13] Kajita S. *et al.* Surface modification of titanium using he plasma. Applied Surface Science 303, 438–445 (2014).

[b14] Kajita S. *et al.* Helium plasma implantation on metals: Nanostructure formation and visible-light photocatalytic response. Journal of Applied Physics 113, 134301 (2013).

[b15] Tanyeli I., Marot L., van de Sanden M. C. M. & Temmerman G. De.. Nanostructuring of iron surfaces by low-energy helium ions. ACS Applied Materials & Interfaces 6, 3462–3468 (2014). 24490884 10.1021/am405624v

[b16] Takamura S. & Uesugi Y.. Experimental identification for physical mechanism of fiber-form nanostructure growth on metal surfaces with helium plasma irradiation. Applied Surface Science 356, 888–897 (2015).

[b17] Fiflis P., Christenson M., Connolly N. & Ruzic D.. Nanostructuring of palladium with low-temperature helium plasma. Nanomaterials 5, 2007 (2015). 10.3390/nano5042007 PMC5304795 28347109

[b18] Novakowski T., Tripathi J. & Hassanein A.. Temperature-dependent surface modification of ta due to high-flux, low-energy He^+^ ion irradiation. Journal of Nuclear Materials 467, Part 1, 244–250 (2015).

[b19] Novakowski T., Tripathi J., Hosinski G., Joseph G. & Hassanein A.. Temperature-dependent surface porosity of Nb_2_O_5_ under high-flux, low-energy he^+^ ion irradiation. Applied Surface Science 362, 35–41 (2016).

[b20] Tanyeli I., Marot L., Mathys D., van de Sanden M. C. M. & Temmerman G. D.. Surface modifications induced by high fluxes of low energy helium ions. Scientific Reports 5, 9779 (2015). 25919912 10.1038/srep09779 PMC4412079

[b21] Kajita S., Saeki T., Yoshida N., Ohno N. & Iwamae A.. Nanostructured black metal: Novel fabrication method by use of self-growing helium bubbles. Applied Physics Express 3, 085204 (2010).

[b22] Yajima M. *et al.* Tritium retention in nanostructured tungsten with large effective surface area. Journal of Nuclear Materials 438, S1142–S1145 (2013).

[b23] de Respinis M. *et al.* Efficient plasma route to nanostructure materials: Case study on the use of m-wo_3_ for solar water splitting. ACS Applied Materials & Interfaces 5, 7621–7625 (2013). 23855799 10.1021/am401936q

[b24] Komori K. *et al.* Sulfur K-edge XANES for methylene blue in photocatalytic reaction over WO_3_ nanomaterials. Nuclear Instruments and Methods in Physics Research Section B: Beam Interactions with Materials and Atoms 365, Part A, 35–38 (2015).

[b25] Iyyakkunnel S. *et al.* Morphological changes of tungsten surfaces by low-flux helium plasma treatment and helium incorporation via magnetron sputtering. ACS Applied Materials & Interfaces 6, 11609–11616 (2014). 24960311 10.1021/am502370t

[b26] Petty T. & Bradley J.. Tungsten nanostructure formation in a magnetron sputtering device. Journal of Nuclear Materials 453, 320–322 (2014).

[b27] Kajita S., Yoshida N., Ohno N. & Tsuji Y.. Growth of multifractal tungsten nanostructure by he bubble induced directional swelling. New Journal of Physics 17, 043038 (2015).

[b28] Shu W., Wakai E. & Yamanishi T.. Blister bursting and deuterium bursting release from tungsten exposed to high fluences of high flux and low energy deuterium plasma. Nucl. Fusion 47, 201–209 (2007).

[b29] Kajita S., Ohno N., Yajima M. & Kato J.. Growth annealing equilibrium of tungsten nanostructures by helium plasma irradiation in non-eroding regimes. Journal of Nuclear Materials 440, 55–62 (2013).

[b30] Tokitani M. *et al.* Desorption of helium from austenitic stainless steel heavily bombarded by low energy He ions. Journal of Nuclear Materials 329–333, 761–765 (2004).

[b31] Behrisch R. & Eckstein W.. Sputtering by particle bombardment: Experiments and Computer Calculations from Threshold to MeV Energies Springer, pp 33–177 2007).

[b32] Noiri Y., Kajita S. & Ohno N.. Nanostructure growth by helium plasma irradiation to tungsten in sputtering regime. Journal of Nuclear Materials 463, 285–288 (2015).

[b33] Kohara S.. Kinzoku Zairyo Gairon, Asakura (*in Japanese*) (1991).

[b34] Plansee group web page. http://www.plansee.com/en/materials/tungsten.html (Date of access: 27/05/2016).

[b35] Tietz T. & Wilson J.. Behavior and properties of refractory metals. Stanford Univ. Press, 1965).

[b36] Ketova V. P. *et al.* Effect of alloying on the modulus of elasticity of platinum alloys. Metal Science and Heat Treatment 12, 615–617 (1970).

[b37] Eckstein W.. Calculated sputtering, reflection and range values, IPP-report 9/132 (2002).

[b38] Smirnov R. & Krasheninnikov S.. On the shear strength of tungsten nano-structures with embedded helium. Nuclear Fusion 53, 082002 (2013).

